# Nucleosome-binding protein HMGN2 exhibits antitumor activity in oral squamous cell carcinoma

**DOI:** 10.3892/ol.2013.1665

**Published:** 2013-11-07

**Authors:** ANKANG HU, XIAOQIAN DONG, XIQIAN LIU, PING ZHANG, YONGHONG ZHANG, NING SU, QIANMING CHEN, YUN FENG

**Affiliations:** State Key Laboratory of Oral Diseases, Sichuan University, Chengdu, Sichuan 610041, P.R. China

**Keywords:** HMGN2, oral squamous cell carcinoma, apoptosis, antitumor activity, cell cycle

## Abstract

Natural killer (NK) cells and cytolytic T lymphocytes (CTLs) serve as effectors in the antitumor response. High mobility group nucleosomal binding domain 2 (HMGN2) is a candidate effector molecule involved in CTL and NK cell function. In the current study, recombinant human HMGN2 was isolated and purified from transformed *Escherichia coli*. Tca8113 cells, an oral squamous cell carcinoma line, were treated with a variety of HMGN2 protein concentrations and cell growth was analyzed. HMGN2 significantly inhibited the growth of Tca8113 cells and was predicted to arrest cells in the S phase. Moreover, HMGN2 treatment increased the apoptosis rate of Tca8113 cells. Western blotting indicated the upregulation of p53 and Bax proteins, whereas Bcl-2 was significantly downregulated. In addition, caspase-3 was found to be activated. Furthermore, the HMGN2 protein may suppress the growth of Tca8113 cells *in vivo*. The results of the current study indicated that the HMGN2 protein may inhibit the growth of oral squamous cell carcinoma and HMGN2 may represent an antitumor effector molecule of CTL or NK cells.

## Introduction

Natural killer (NK) cells and cytolytic T lymphocytes (CTLs) function as antitumor immune effectors. NK cells and CTLs are rich in cytoplasmic granules. Upon degranulation, these cells release cytotoxic substances that act on target cells (1). Granules in the cytoplasm of CTL contain perforin, granzyme, granulysin, additional effector molecules involved in the antitumor response and several uncharacterized components (2,3).

In our previous study, an antimicrobial polypeptide was isolated and purified from interleukin (IL)-2-stimulated human peripheral blood mononuclear leukocytes and the polypeptide was identified as high mobility group nucleosomal binding domain 2 (HMGN2). Mononuclear leukocytes were stimulated with IL-2 *in vitro* and it was confirmed that HMGN2 is expressed in the cytoplasm and is secreted. Therefore, HMGN2 may represent a candidate effector molecule involved in CTL and NK cell function (4). HMGN2 is one of the most abundant non-histone nuclear proteins of vertebrates and invertebrates (5). The HMGN2 gene is highly conserved and is located near several tumor suppressor genes (6,7). In addition, the α-helical domain of HMGN2 homes to tumors, particularly to their vascular endothelia (8).

In the present study, recombinant human HMGN2 protein was prepared and its antitumor activity was examined in the oral squamous cell carcinoma cell line, Tca8113.

## Materials and methods

### Production and isolation of recombinant human HMGN2 protein

Total RNA was isolated from stimulated mononuclear leukocytes using TRIzol reagent (Gibco-BRL, Carlsbad, CA, USA). The full-length HMGN2 cDNA was amplified by reverse transcription-polymerase chain reaction (RT-PCR) and was ligated into a pGEX-4T-1 expression vector. The following primers were designed and prepared: P1, 5′-ACG GAT CCC CCA AGA GAA AGG CTG-3′ and P2, 5′-TAG AAT TCC TTG GCA TCC TCC AGC AC-3′, containing *Bam*HI and *Xho*I restriction sites, respectively. The HMGN2 insert in pGEX-HMGN2 was sequence-verified. *Escherichia coli* BL21 (State Key Laboratory of Oral Disease, Sichuan University, Chengdu, China) were transformed with pGEX-HMGN2. Cells were cultured in Luria-Bertani medium for 12 h in the presence of isopropylthio-β-D-galactoside (Sigma-Aldrich, St. Louis, MO, USA) to induce protein expression. Induced *E. coli* were washed with phosphate-buffered saline (PBS) and cell lysates were prepared with five freeze/thaw cycles in the presence of lysozyme. Following centrifugation at 10,000 × g for 10 min, glutathione *S*-transferase (GST)-HMGN2 fusion proteins were purified from supernatants using a Glutathione Sepharose 4B column (Amersham Pharmacia Biotech, Amersham, UK). Purified fusion proteins were cleaved by thrombin digestion. The recombinant HMGN2 protein was recovered by reverse-phase high-performance liquid chromatography (RP-HPLC). Protein concentrations were measured using a bicinchoninic acid (BCA) protein assay kit (Pierce Biotechnology, Inc., Rockford, IL, USA) with bovine serum albumin (BSA) as the standard.

### Cell culture

Tca8113 cells were obtained from the State Key Laboratory of Oral Disease (Sichuan University, Chengdu, China). Cells were cultured in RPMI-1640 medium (Gibco-BRL) supplemented with 10% fetal bovine serum (FBS; Gibco-BRL), 100 U/ml penicillin and streptomycin in a humidified incubator at 37°C with 5% CO_2_.

### MTT assay

Tca8113 cells were plated in 96-well plates at a density of 1×10^4^ cells/well. Cells were treated with various concentrations of HMGN2 protein (0, 1, 2, 3, 4 and 5 μg/ml) for 48 h. Following this, the medium was replaced in each well with 200 μl fresh medium containing MTT [2.5 mg dissolved in 50 μl dimethylsulfoxide (DMSO)]. Following incubation for 4 h at 37°C, the MTT medium in each well was replaced with 100 μl DMSO. Viable cells were detected by measuring the absorbance at 570 nm.

### Apoptosis assay

Tca8113 cells were seeded at a density of 5×10^5^ cells/well in six-well plates and incubated overnight. Medium was replaced with maintenance medium containing the appointed concentration of HGMN2 protein and cells were incubated for 24 h. Cells were harvested by trypsin digestion and stained using the Annexin V-FITC apoptosis detection kit (R&D Systems, Minneapolis, MN, USA), according to the manufacturer’s instructions. Briefly, pretreated Tca8113 cells were washed twice with FCM buffer (PBS with 5% FBS and 0.1% NaN_3_). Next, cells were incubated with Annexin V-FITC for 30 min at 4°C. Propidium iodide (PI; 50 μg/ml) was added and cells were analyzed by a Beckman Coulter FC500 with submit 5.2 software (Beckman Coulter, Miami, FL, USA).

Apoptosis was measured using Hoechst 33258 (Promega Corporation, Madison, WI, USA), according to the manufacturer’s instructions. Cells were cultured overnight in six-well plates and treated with HMGN2 protein for 24 h. Subsequently, cells were washed with PBS and fixed with a solution of 4% methanol-free formaldehyde in PBS for 25 min. Staining was performed according to the manufacturer’s instructions. Fluorescence was visualized using an Olympus BX60 microscope (Olympus Corporation, Tokyo, Japan).

### Cell cycle analysis

Tca8113 cells were seeded at a density of 5×10^5^ cells/well in six-well plates. Cells were incubated overnight and the medium was replaced with maintenance medium containing the appointed concentration of HGMN2 protein. After 24 h, floating and trypsin-harvested cells were combined and cell cycles were analyzed using PI staining. Briefly, cells were washed with cold PBS, fixed with cold 70% ethanol and maintained at 4°C overnight. Cells were then washed once with PBS, digested with 200 μl RNase (1 mg/ml) at 37°C for 30 min, and stained with 800 μl PI (50 μg/ml) at room temperature for 30 min. Cells were analyzed using a Beckman Coulter FC500 with submit 5.2 software. Cell cycle histograms were analyzed using MultiCycle for Windows software (Beckman Coulter).

### Western blotting

Total proteins were isolated from cultured cells using a total protein extraction kit (Nanjing KeyGen Biotech. Co. Ltd., Nanjing, China) and protein concentrations were measured using a BCA protein assay kit (Pierce Biotechnology, Inc.). Proteins were separated by 15% SDS-PAGE and transferred electrophoretically to polyvinylidene difluoride membranes (Millipore, Billerica, MA, USA). Membranes were blocked with 2% BSA in TBS containing 0.1% Tween 20 (TBST) for 2 h at 37°C. Next, membranes were incubated for 2 h in anti-p53 (polyclonal/rabbit), anti-Bax (polyclonal/rabbit), anti-Bcl-2 (polyclonal/rabbit), anti-caspase-3 (polyclonal/rabbit) and anti-GAPDH (monoclonal/mouse) (all 1:500; Cell Signaling Technology, Inc., Beverly, MA, USA). Membranes were subsequently exposed to horseradish peroxidase-conjugated anti-mouse or -rabbit IgG secondary antibodies (1:5,000 in TBST with 2% BSA) for 1 h at 37°C. Proteins were quantified by band densitometry (GS-700; Bio-Rad, Hercules, CA, USA) using Quantity One 4.4.0 software (Bio-Rad).

### In vivo tumor formation assay

Nude mice were randomly divided into three groups of seven mice each. Tca8113 cells (1×10^7^) were injected subcutaneously to construct a xenotransplantation tumor model. HMGN2 (50 ng/g weight) was injected around the tumor tissue on days 21, 25, 29 and 33 following tumor cell transplantation. Identical doses of cisplatin and PBS were used as positive and negative controls, respectively. All nude mice were sacrificed at post-transplantation day 37 and images were captured (Nikon J1, Wuxi, China). Tumor tissue was removed and the tumor volume was calculated as follows: Tumor volume = 1/2 × (longer diameter) × (shorter diameter)^2^.

### Hematoxylin and eosin (H&E) staining

Tca8113 xenograft specimens were fixed in 10% buffered formalin, processed and embedded in paraffin. Sections (3-μm thick) were cut and stained with H&E. Slides were visualized using an Imager Z1 microscope equipped with an AxioCam MRc5 camera (Carl Zeiss AG, Oberkochen, Germany).

### Statistical analysis

One-tailed unpaired Student’s t-tests were used to detect significant differences among treatment groups. P<0.05 was considered to indicate a statistically significant difference.

## Results

### Purification and characterization of recombinant human HMGN2 protein

**Figure 1:**
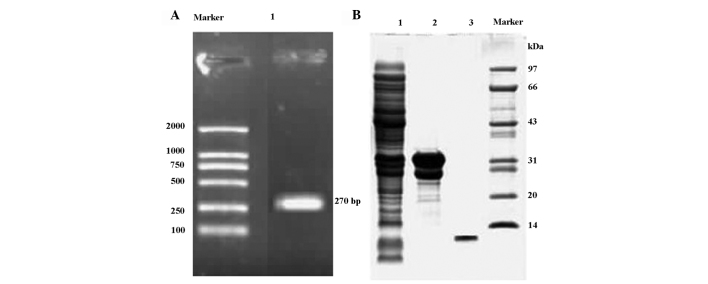
(A) cDNA fragments corresponding to HMGN2. (B) SDS-PAGE was used to identify recombinant HMGN2. Lanes 1, total protein extracted from *Escherichia coli* and transformed with pGEX-HMGN2; 2 and 3, purified glutathione *S*-transferase-HMGN2 fusion proteins and recombinant HMGN2, respectively. HMGN2, high mobility group nucleosomal binding domain 2.

Holo-HMGN2 cDNA was prepared by RT-PCR (Fig. 1A) and used to construct the prokaryotic expression vector, pGEX-HMGN2. The insert sequence and orientation of the recombinant vector were confirmed by direct sequencing. *E. coli* BL21, transformed with the pGEX-HMGN2 construct, generated HMGN2 fusion proteins in bulk that were purified by GST affinity chromatography. Purified recombinant HMGN2 was obtained using RP-HPLC (Fig. 1B).

### HMGN2 inhibits the growth and colony formation of Tca8113 cells

**Figure 2:**
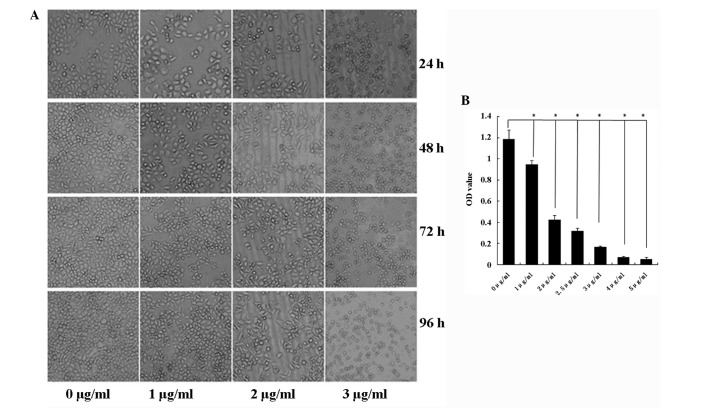
HMGN2 protein inhibits cell growth and colony formation ability of Tca8113 cells. (A) Effect of HMGN2 treatment on Tca8113 cells and (B) cell proliferation by MTT assay. Data are presented as the mean ± SEM (n=3). ^*^P<0.001, vs. 0 μg/ml control. HMGN2, high mobility group nucleosomal binding domain 2.

Tca8113 cell growth was suppressed in response to HMGN2 treatment (Fig. 2A). The MTT assay was used to assess the toxicity of HMGN2 expression in Tca8113 cells. At HMGN2 protein concentrations of 1, 2, 3, 4 and 5 μg/ml, Tca8113 cell growth decreased by ~20, 70, 80, 90 and 95%, respectively (Fig. 2B).

### HMGN2 induces S phase cell cycle arrest in Tca8113 cells

**Figure 3:**
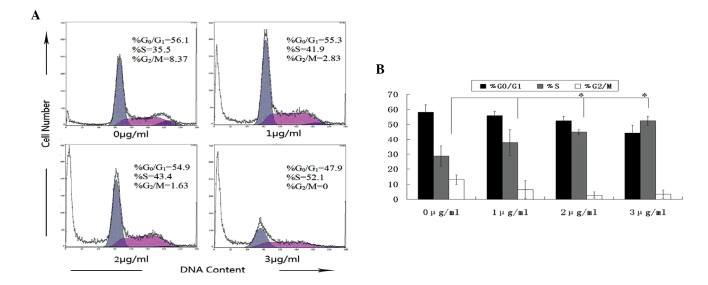
(A) Flow cytometry of cell cycle distribution at 24-h post-treatment. (B) HMGN2 protein induces S phase cell cycle arrest in Tca8113 cells. Data are presented as the mean ± SEM (n=3). ^*^P<0.05, vs. 0 μg/ml control. HMGN2, high mobility group nucleosomal binding domain 2.

To investigate the potential mechanisms by which HMGN2 inhibits Tca8113 cell growth, the effect of HMGN2 on the cell cycle was evaluated by flow cytometry. At 24-h post-treatment, the percentage of untreated Tca8113 cells in S phase was 35.5%, whereas the percentage of Tca8113 cells exposed to 3 μg/ml HMGN2 in S phase was 52.1% (Fig. 3). In addition, 10–15% of untreated Tca8113 cells were in the G2/M phase compared with 5% of cells treated with 3 μg/ml HMGN2. These results indicate that HMGN2 treatment may arrest Tca8113 cells in S phase by inhibiting the S-G2 transition.

### HMGN2 induces apoptosis in Tca8113 cells

**Figure 4:**
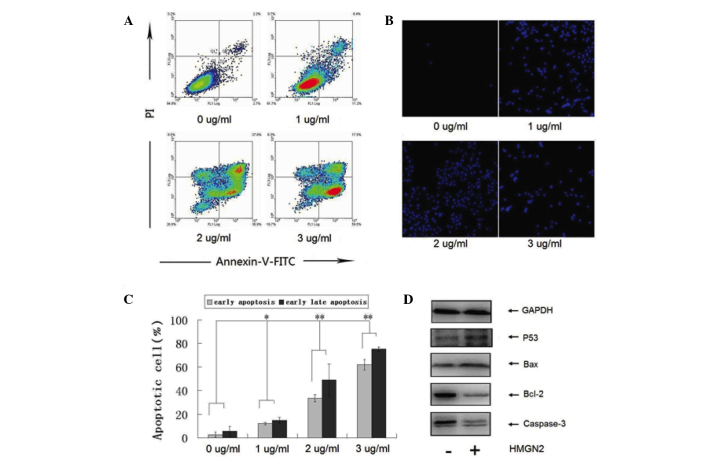
HMGN2 protein induces apoptosis in Tca8113 cells. (A and B) Quantitative analysis of cell apoptosis measured by Annexin V and PI double staining. (C) Examination of apoptosis by Hoechst assay. (D) Effect of HMGN2 treatment on the expression of GAPDH, p53, Bax, Bcl-2 and caspase-3. Data are presented as mean ± SEM for triplicate analyses. ^*^P<0.05 and ^**^P<0.01, vs. 0 μg/ml control. HMGN2, high mobility group nucleosomal binding domain 2.

To investigate whether the HMGN2-induced growth inhibition of Tca8113 cells is associated with apoptosis, HMGN2-exposed cells were analyzed by flow cytometry and fluorescent microscopy following staining with Annexin V/PI or Hoechst. The results indicated that HMGN2 induced Tca8113 cell apoptosis in a dose-dependent manner (Figs. 4A and B). Compared with untreated cells, the percentage of apoptotic cells (Annexin V^+^/PI^−^ and Annexin V^+^/PI^+^) was significantly increased following exposure to >1 μg/ml HMGN2. The percentage of apoptotic cells exposed to concentrations of 0, 1, 2 and 3 μg/ml HMGN2 protein were 5, 18, 65 and 77%, respectively. Consistent with the Annexin V/PI double staining results, the number of Hoechst-positive cells examined by fluorescence microscopy was also significantly increased following treatment with >1 μg/ml HMGN2 (Fig. 4C). Next, the effects of HMGN2 treatment on the expression of p53, Bcl-2, Bax and caspase-3 were examined. When Tca8113 cells were exposed to 2 μg/ml HMGN2 protein for 24 h, the levels of p53 and Bax proteins were upregulated, whereas Bcl-2 was significantly downregulated. In addition, caspase-3 was found to be activated (Fig. 4D).

### HMGN2 suppresses the growth of Tca8113 cells in vivo

**Figure 5:**
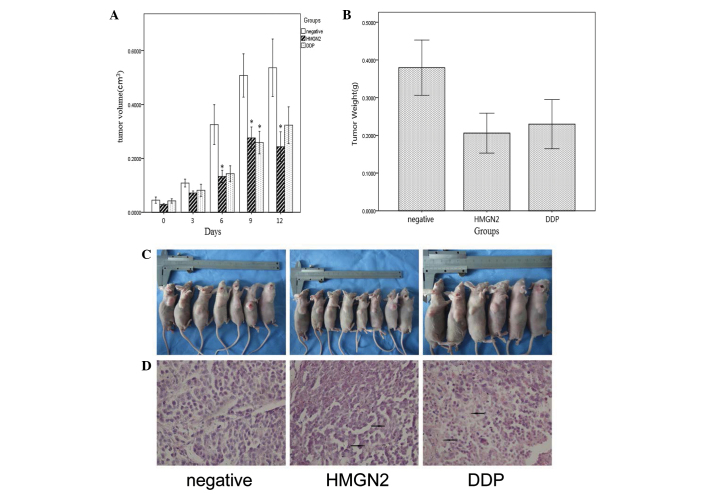
HMGN2 protein suppresses the growth of Tca8113 cell xenografts *in vivo*. (A) Tumor volume following HMGN2 treatment. (B) Final tumor weight at necropsy, 12 days after seeding. Data are presented as mean ± SEM. ^*^P<0.05, vs. negative group. (C) Tca8113 xenografts from each treatment group. HMGN2 protein expression induced necrosis in Tca8113-xenografted tumor tissues. (D) Paraffin-embedded sections of representative Tca8113 xenografts analyzed by hematoxylin and eosin staining. Arrows indicate necrotic tissue. HMGN2, high mobility group nucleosomal binding domain 2.

A tumor formation assay was performed to determine whether HMGN2 is able to affect the growth of Tca8113 cells *in vivo*. The growth rate of xenografts in HMGN2-treated groups was slower compared with that of untreated controls, particularly during the initial 20 days. A 50% reduction in average tumor volume was observed in HMGN2-treated tumors compared with controls. Upon completion of the experiment, the average weight of tumors excised from HMGN2-treated animals was ~40% of the average control weight (Fig. 5). H&E staining indicated that necrosis occurred in the majority of the HMGN2-treated xenografts during tumor formation.

## Discussion

HMG proteins have been described as an abundant family of non-histone proteins in the cell nucleus of vertebrate and invertebrate organisms (9). The HMG protein family is subdivided into three subfamilies: HMGB, HMGA and HMGN. Each subfamily appears to exert a single characteristic nuclear function (9), however, peptides in the HMG protein family also exhibit adjunct roles. For example, HMG box1 (HMGB1) is an abundant, highly conserved cellular protein, widely known as a nuclear DNA-binding protein, which stabilizes nucleosome formation, facilitates gene transcription and regulates the activity of a steroid hormone receptor (10,11). A decade-long search has culminated in HMGB1 as a late toxic cytokine of endotoxemia. HMGB1, released by macrophages upon exposure to endotoxins, activates a number of other proinflammatory mediators and is lethal to otherwise healthy animals (10,11). In addition, HMGB1 exhibits potent bactericidal activity (12). Fernandes *et al*(13) identified an HMG family peptide in the mucus secretions of fish skin that also exhibits potent antimicrobial activity.

The HMGN family includes five chromatin architectural proteins that are present in higher vertebrates (14). Of these proteins, HMGN1, 2 and 4 are expressed ubiquitously (15,16), whereas HMGN3 and 5 are expressed in specific tissues (17,18). Initially, HMGNs were regarded as transcription coregulators, however, their roles in DNA repair and cancer progression have been determined using HMGN1 knockout mice (19). These studies indicate that the archetype of HMGN1 exhibits characteristics of a tumor suppressor gene. In addition to HMGN1, the expression of HMGN5 (formerly NSBP1) (20) was found to be elevated four-fold in highly metastatic breast cancer cells compared with that in low metastatic cells (21). In mice, overexpression of HMGN5 in the uterus was associated with the development of uterine adenocarcinoma (22). These studies are consistent with the involvement of HMGN5 in cancer progression.

The HMGN2 gene is located on chromosome 1p36.1 and contains six exons (23) with an extremely high GC content and an ‘*Hpa*II tiny fragment’ island. These hallmarks are indicative of a housekeeping gene that may be crucial to the basal functioning of cells (7,8). However, biological roles of this protein have been poorly defined. HMGN2 is preferentially associated with chromatin subunits (9), and abnormal HMGN2 gene or protein expression is associated with neoplasms and autoimmune diseases (24,25). Porkka *et al*(8) examined phage-displayed cDNA libraries *in vivo* to identify phages capable of homing to the vascular endothelia of tumors. The screen revealed a markedly potent homing peptide, F3, which corresponded to a 17- to 48-aa fragment in HMGN2. The 31-residue peptide selectively bound tumor cells *in vitro* and *in vivo*.

CTL and NK cells are rich in cytoplasmic granules. Following degranulation, the cells release specific biologically active substances that are cytotoxic to target cells (1). The granules in the cytoplasm of CTL contain perforin, granzyme, granulysin and other effector molecules involved in the antitumor effect, as well as certain unidentified components (2,3). In our previous study (4), HMGN2 was found to be released by human peripheral blood mononuclear leukocytes in the presence of IL-2. HMGN2 may represent an effector molecule for CTL or NK cells.

The present study investigated the activity of the HMGN2 protein in the oral squamous cell carcinoma line, Tca8113. HMGN2 protein was demonstrated to inhibit the growth of Tca8113 cells and partially induce apoptosis. Western blotting indicated the upregulation of p53 and Bax proteins, whereas Bcl-2 was significantly downregulated. In addition, caspase-3 was found to be activated and HMGN2 protein is likely to suppress the growth of Tca8113 cells *in vivo*. The results indicate that HMGN2 protein exhibits antitumor activity against oral squamous cell carcinoma and that HMGN2 may represent a candidate effector molecule for CTL or NK cells. Studies are underway to delineate the role of HMGN2 as an effector molecule for the antitumor activity of CTL or NK cells.
